# Enhanced fluorescence emission or singlet oxygen production of cationic porphyrazines and porphyrins through combination with carbon dots

**DOI:** 10.1111/php.14119

**Published:** 2025-05-21

**Authors:** Gustavo Wander Streit, Rafael Bernardino Rodrigues da Silva Taques, Gabriela Fernandes Barreto, Fabiano Vargas Pereira, Gilson DeFreitas‐Silva, Thiago Teixeira Tasso

**Affiliations:** ^1^ Chemistry Department Institute of Exact Sciences, Universidade Federal de Minas Gerais Belo Horizonte MG Brazil

**Keywords:** carbon dot, fluorescence, porphyrazine, porphyrin, singlet oxygen

## Abstract

A cationic porphyrin and porphyrazine with the 3‐ethylpyridyl substituent (H_2_P and H_2_Pz) and their respective zinc complexes (ZnP and ZnPz) were assembled to a carbon dot (CD) synthesized from citric acid and ammonium citrate. A titration was performed using a fluorescence spectrophotometer to determine the stoichiometric ratio at which the maximum interaction between the substances occurs, as well as the Stern–Volmer constant and intrinsic binding constant. The combination between CD and porphyrins or porphyrazines was confirmed using UV–VIS absorption spectroscopy, fluorescence emission, zeta potential, and Diffusion‐Ordered NMR Spectroscopy (DOSY). It was observed that after combination, there is a decline in the absorption of porphyrin derivatives, a variation in the emission of porphyrazines, a subtle increase in the zeta potential compared to the isolated CD particles, and a variation in the translational diffusion coefficient. It was also found that upon combination with the CD, changes in the photophysical properties of the macrocycles occur, for example, the fluorescence quantum yield of H_2_Pz increases from 0.81 ± 0.03% to 1.97 ± 0.05% while the singlet oxygen quantum yield of H_2_P increases ca. 70%. These results exemplify the capacity of CD to boost some properties of photosensitizers that are key for photodynamic therapy applications.

## Graphical abstract

A cationic free‐base porphyrin and porphyrazine and their zinc(II) complexes were combined to carbon dots (CD). The macrocycles strongly interact with the nanoparticles, quenching the CD fluorescence and enhancing the fluorescence emission efficiency of a porphyrazine by 140% and the singlet oxygen production of a porphyrin by 70%.

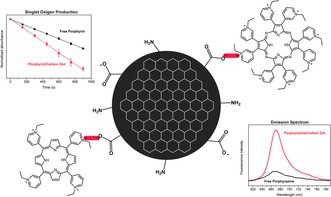

**Abbreviations**
1H: NMR proton nuclear magnetic resonance
ABDA: 9,10‐anthracenediyl‐bismethylenedimalonic acid
CD: carbon dots
DOSY: diffusion‐ordered NMR spectroscopy
FTIR: Fourier transform infrared
HRTEM: high‐resolution transmission electron microscopy
PDT: photodynamic therapy
PS: photosensitizer
ROS: reactive oxygen species
TEM: transmission electron microscopy
UV‐VIS: ultraviolet‐visible region
XRD: X‐ray diffraction

## INTRODUCTION

Photodynamic therapy (PDT) has emerged as an alternative treatment for conditions ranging from age‐related macular degeneration to different types of cancers, including diseases caused by bacteria, fungi, and protozoa.^1^, ^2^, ^3^ The success of PDT relies, among other aspects, on the spatial control of the treated area, which is achieved by the selective activation of the drug's active ingredient, named photosensitizer (PS), by a light source. As a result, PDT is established as a minimally invasive treatment with reduced side effects for the patient.^4^, ^5^

PDT mechanism of action is based on the destruction of cells and targeted tissues by the attack of reactive oxygen species (ROS) produced by the PS. Excitation of the PS with visible light leads to its transition to a triplet state which can: (i) interact with molecular oxygen (^3^O_2_) via energy transfer, producing singlet oxygen (^1^O_2_), and/or (ii) engage in hydrogen atom or electron transfer reactions with surrounding molecules, producing radical species that result in other ROS, such as hydroxyl and peroxyl radicals, hydrogen peroxide, among others.^6^, ^7^ In this sense, PSs are strong visible‐light absorbing molecules with efficient ROS production. Porphyrins and their derivatives, such as chlorins, are a well‐known class of PSs with several examples in clinical practice and under clinical trials all over the world.^5^, ^8^ The association of these macrocycles with nanostructures, such as liposomes,^9^, ^10^ inorganic nanoparticles,^11^, ^12^ and carbon‐based materials,^13^, ^14^ for instance, are strategies used to potentialize several properties of these PSs, including ROS production, fluorescence emission efficiency, tumor specificity, cellular uptake and so on. Carbon dots (CD) are nanoparticles smaller than 10 nm with a carbogenic core, whose structure, whether amorphous or crystalline, depends on the synthesis method and precursor materials.^15^ The surface of the CD can have different functional groups (eg. –OH, NH_2_, and –COOH), depending on the precursors used for the synthesis and any chemical treatments performed on the nanoparticles. Thus, the class of CD is quite extensive in terms of properties due to the wide variety of starting materials and methods used to synthesize these nanoparticles. Despite this diversity, many CD produced from suitable precursors exhibit low toxicity and high biocompatibility, particularly those smaller than 6 nm, as they can be rapidly excreted from the body through renal clearance.^16^, ^17^ As a result, CD have emerged as materials with promising properties for biological applications, such as biocompatibility, high water solubility, chemical inertness, strong fluorescence emission, and high versatility.^18^, ^19^ In this context, CD have been used alone or combined with agents, including drugs, antibodies, and genes for imaging and therapy applications.^20^

**SCHEME 1:**
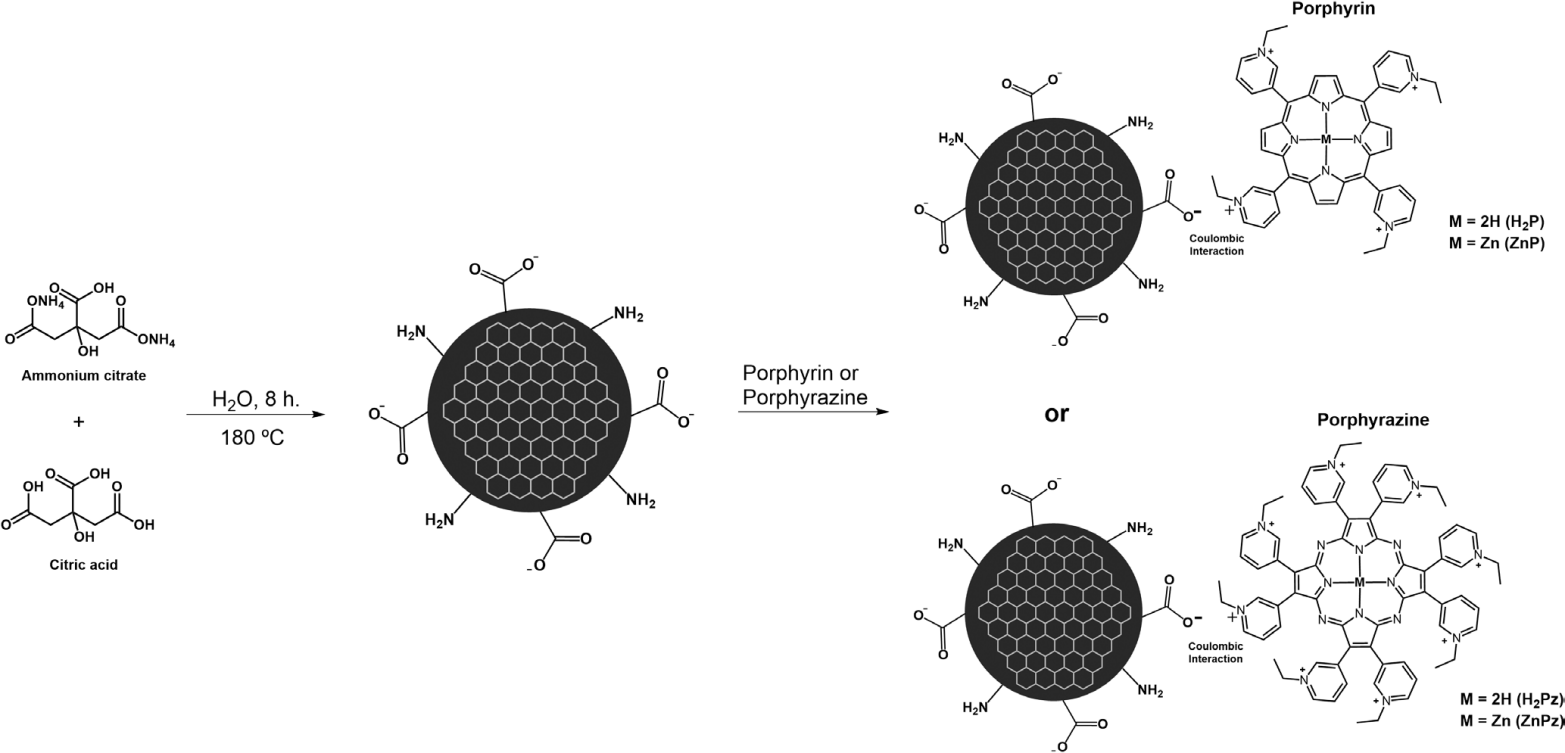
Synthesis of carbon dot and structure of the porphyrins and porphyrazines.

Recently, the combination of CD with PSs, namely porphyrins and chlorins, has been investigated to enhance their photophysical and photodynamic responses in tumor cells. The aggregates often exhibit stronger fluorescence emission (due to resonance energy transfer from the CD), higher photostability, increased production of singlet oxygen, and higher phototoxicity compared to non‐aggregates PSs.^21^, ^22^, ^23^, ^24^ In our group, we have recently studied the photoactivity of cationic porphyrins and porphyrazines (a porphyrin analogue) against skin cell lines, verifying that the wavelength of the irradiation source could modulate their phototoxicity and selectivity toward the cancer cells.^25^ Considering these results, we seek to further enhance the applicability of these PSs for PDT by associating them with CD. In this context, this work aims to investigate the effect of CD (from ammonium citrate and citric acid) assembly on the fluorescence and singlet oxygen production of cationic porphyrins and porphyrazines ligands and their complexes with zinc(II) (Scheme 1). To the best of our knowledge, this is the first work to report the interaction of CD with porphyrazines.

## MATERIALS AND METHODS

### Materials

Citric acid and 9,10‐anthracenediyl‐bismethylenedimalonic acid (ABDA, ≥90%) were acquired from Sigma Aldrich (St. Louis, USA), whereas ammonium citrate was purchased from Synth (São Paulo, Brazil). The reagents were used without further purification. The porphyrins and porphyrazines (referred in the text as porphyrin derivatives) were obtained as described in previous work by our group.^25^

### Preparation of carbon dots

The carbon dot (CD) was prepared using hydrothermal carbonization in an autoclave reactor. Initially, 15 mmol of citric acid and the same amount of ammonium citrate were dissolved in 30 mL of deionized water. The solution was stirred on a magnetic stirrer to form a clear solution. The solution was then transferred to a Teflon‐lined stainless‐steel autoclave and heated at 180°C for 8 h. Afterward, the solution was centrifuged to remove any precipitate, and the supernatant was filtered through a 0.22 μm pore size PTFE syringe filter (Filtrilo). The filtered material was dried in a vacuum oven for 72 h at 100°C.

### Characterization methods

The UV–VIS absorption spectra were measured using a Hitachi U‐2010 and an HP 8453A diode‐array spectrophotometer, using quartz cuvettes with a 1.0 cm path length.

Zeta potential measurements were performed in aqueous solutions of the CD assemblies (pH within the range 2.93–3.60) using Zetasizer Nano ZS equipment from Malvern Instruments (Malvern, UK).

The photoluminescence spectra were obtained with a Varian Cary Eclipse spectrometer at various excitation wavelengths.

Fourier transform infrared (FTIR) spectroscopy of the samples was performed using a Perkin Elmer Spectrum RXI spectrometer employing the attenuated total reflection (ATR) technique.

Transmission electron microscopy (TEM) images of the CD were acquired using a Tecnai G2‐20 SuperTwin FEI (200 kV). Diluted suspensions of the carbon nanoparticles were deposited onto an ultrathin carbon film for imaging. By measuring the size of CDs from various images (using ImageJ software), the average size and size distribution of the nanoparticles were determined based on measurements of 100 isolated nanoparticles. Some of the images used to determine the size distribution are included in the supporting information (Figure S1).

Raman spectra of the samples were obtained using a Witec alpha300 RA spectrophotometer with a 523 nm wavelength laser. For sample preparation, a drop of the CD suspension was placed onto a glass slide and allowed to dry at room temperature.

X‐ray diffraction (XRD) analysis was performed on a dried sample of the CD using an Anton Paar XRDynanic‐500, operated at 40 kV and 50 mA. Scans were conducted in the 2θ range of 5–80° at a scan rate of 0.01°s^−1^.

Proton nuclear magnetic resonance (^1^H NMR) spectra were obtained in deuterium oxide (D_2_O) for pure H_2_P, pure CD, and H_2_P/CD using a Bruker Avance DRX 600 spectrometer. The ^1^H NMR analyses were performed in 32 scans with pre‐saturation of the water signal in D_2_O (4.70 ppm). The Diffusion‐Ordered NMR Spectroscopy (DOSY) experiments were performed using the stebpgp1s19 pulse sequence, with the following parameters: number of scans = 32, number of acquired points = 4 k, number of diffusion points = 16, and diffusion curve type = linear.

### Calibration curve for determination of CD concentration

In a quartz cuvette with four polished sides, 3 mL of distilled water was added and the temperature was allowed to reach 25°C. Then, 10 μL aliquots of a 21.7 g L^−1^ CD stock solution were added to the cuvette, under magnetic stirring, up to a total of 90 μL. The emission spectrum was obtained from 320 to 600 nm with excitation at 310 nm after each aliquot addition (Figure S2). A concentration versus fluorescence intensity curve was plotted, and the slope was designated as *κ*.

### Carbon dot titration with porphyrin derivatives

In a quartz cuvette with four polished faces, under magnetic stirring, 3 mL of distilled water and 50 μL of a CD stock solution (21.7 g L^−1^) were added. The system temperature was allowed to reach 25°C, and the emission spectrum was obtained by exciting at 310 nm and analyzing the region from 320 to 600 nm. The titration was performed by adding aliquots of a stock solution (1 × 10^−3^ mol L^−1^) of the porphyrin derivative until the decay of the CD emission band intensity was no longer observed. Since the porphyrin derivatives absorb light in the CD emission region, a filter effect experiment was performed using UV–VIS spectroscopy, repeating the additions made during the titration, but without the carbon dot. The absorbance values at 310 and 391 nm were used in Equation (1)^26^ to obtain the corrected fluorescence intensity:

(1) $$\;F=F_{\mathrm{obs}}\times \;10^{\frac{A_{\mathrm{ex}}+A_{\mathrm{em}}}{2}}$$

where *F*_obs_ is the observed fluorescence intensity (without correction), *A*_ex_ is the absorbance at the excitation wavelength, and *A*_em_ is the absorbance at the emission wavelength.

A concentration versus corrected fluorescence intensity curve was plotted (Figure S3) to verify the endpoint of the titration, allowing for the determination of the stoichiometric ratio at which maximum interaction between the substances occurs. The Stern–Volmer constant (*K*_SV_) was obtained from the slope of (*F*_0_ − *F*)/*F* versus quencher concentration plot (Figure S4), according to Equation (2)^26^:

(2) $$\frac{F_{0}-F}{F}=K_{\mathrm{sv}}\left[Q\right]$$

where *F*_0_ is the initial fluorescence intensity, *F* is the fluorescence intensity measured after addition of the substance corrected with the filter effect, and *Q* is the concentration of the added substance. The corrected fluorescence intensity (*F*) was calculated from Equation (1).^26^

The intrinsic binding constant (*K*_b_) was obtained from the plot of log((*F*_0_ − *F*)/*F*) versus concentration (Figure S5), according to Equation (3)^26^:

(3) $$\log\left(\frac{F_{0}-F}{F}\right)=\log K_{b}+n\log\left[Q\right]$$

where *Q* is the concentration of the porphyrin derivatives and *n* is the Hill coefficient. The values of *n* obtained for H_2_P, ZnP, H_2_Pz, and ZnPz were equal to 1.10, 1.03, 0.96, and 1.90, respectively.

### Temperature effect on CD combination with the porphyrin derivatives

In a quartz cuvette, under magnetic stirring, 3 mL of distilled water and 50 μL of a CD stock solution (21.7 g L^−1^) were added. The system temperature was allowed to reach 10°C, and the emission spectrum of the solution was obtained by exciting at 310 nm and analyzing the region from 320 to 600 nm. A spectrum was recorded after each addition of 5 μL of the porphyrin derivative up to a total of 40 μL. This procedure was repeated at temperatures of 20 and 30°C, and a graph of concentration versus F_0_/F was plotted (Figure S6).

### Fluorescence quantum yield ( *Φ*_F_ ) determination

The experiment to determine the fluorescence quantum yield (*Φ*_F_) of the free and combined porphyrin derivatives was based on previously reported procedures.^27^, ^28^ Each sample was measured in triplicate, using cresyl violet (*Φ*_F_ = 0.40 in water^29^) and quinine sulfate (*Φ*_F_ = 0.54 in 0.1 mol L^−1^ sulfuric acid^30^) as references. In a cuvette, the absorbance and the emission spectra of each sample in four different concentrations were measured in water. The free and combined porphyrins were excited at 577 nm and their emission spectra were integrated from 590 to 800 nm, while the free and combined porphyrazines were excited at 590 nm and their spectra were integrated from 600 to 800 nm. Non‐combined CD was excited at 340 nm and measured from 350 to 600 nm. The Equation (4)^27^, ^28^ was used to determine *Φ*_F_:

(4) $$\Phi_{\mathrm{Fs}}=\Phi_{\mathrm{Fr}}\times \left(\frac{T_{s}}{T_{r}}\right)\times \left(\frac{\eta_{s}^{2}}{\eta_{r}^{2}}\;\right)$$

where *T* refers to the slope of the linear interpolation of the graph of absorbance versus the area of the emission spectrum, *η* is the refractive index of the solvent, and s and r represent the substance of interest and the reference substance, respectively.

### Fluorescence lifetimes

Fluorescence time‐course measurements were recorded by time‐correlated single photon counting on a picosecond laser spectrometer (Edinburgh FL900 L‐Format spectrometer with monochromator in the emission channel) using a mode‐locked Ti:sapphire laser (Tsunami 3950 pumped by Millennia × Spectra Physics) as the source producing 1 picosecond FWHM pulses with a repetition rate of 8 MHz (Spectra Physics 3980 pulse selector). The laser wavelength was adjusted with second harmonic generators to produce excitation pulses at 298 nm for the sample. Single photon detection was performed using a Hamamatsu R3809U cooled microchannel plate photomultiplier. Lifetime decay spectra can be seen in the supporting information (Figure S7) and the average lifetimes (*τ*_ave_) were determined from the weighted average of the results obtained from *τ*_1_, *τ*_2_, *τ*_3_ and the respective amplitudes *A*_1_, *A*_2_, and *A*_3_.

### Singlet oxygen quantum yield ( *Φ*_Δ_ ) determination

Quantification of singlet oxygen production was performed for the free and combined porphyrin derivatives by the indirect method using absorption spectroscopy in the UV–VIS region, employing ABDA as a probe. Methylene blue (*Φ*_Δ_ = 0.60 in water^31^) was used as a reference substance for irradiation at 648 nm, and rose bengal (*Φ*_Δ_ = 0.75 in water^32^) was used for irradiation at 532 nm. ABDA solutions with an absorbance close to 1.0 at 378 nm were prepared in a cuvette containing 2 mL of water. Solutions of all samples and reference substances were prepared with an absorbance lower than 0.1 at the excitation wavelength. Irradiations were performed at defined time intervals to obtain at least 5 absorption spectra until a 30% decay in the initial absorbance of the probe band was observed. Finally, Equation (5)^33^, ^34^ was used to determine *Φ*_Δ_:

(5) $$\Phi_{\mathrm{\Delta s}}=\Phi_{\mathrm{\Delta r}}\times \left(\frac{S_{s}}{S_{r}}\right)\times \left(\frac{F_{r}}{F_{s}}\right)$$

where *s* and *r* refer to the substances of interest (free and combined porphyrin derivatives) and reference substances (methylene blue and rose bengal), respectively, *S* is the slope of the linear interpolation of the graph of absorbance versus irradiation time, and *F* is the correction factor for the absorbance at the irradiation wavelength (*F* = 1–10^−ABS^).

## RESULTS AND DISCUSSION

### Structural and optical characterization of the carbon dots

The CD was prepared using the method commonly known as “bottom‐up”, in which low molecular weight precursors are employed to form functionalized carbon nanostructures. The formation of the CD starts with polymerization reactions between the precursors, leading to the formation of highly entangled polymer chains.^35^ This is followed by a carbonization process that occurs under high temperatures and pressure, during which the number of polymeric structures decreases as dehydration reactions occur, thereby increasing the relative carbon content in the nanoparticles.^36^ At the end of the CD formation process, approximately spherical nanoparticles are obtained, consisting of a carbon core and a highly functionalized shell. In this work, citric acid served as the source of carbon and oxygen, while ammonium citrate provided carbon, oxygen, and nitrogen atoms in the final structure of the CD. Carbon is essential for forming the primary structure of the nanoparticles, while the nitrogen‐containing functional groups enhance functionalization and improve the fluorescence properties of the nanoparticles.^37^

**FIGURE 1:**
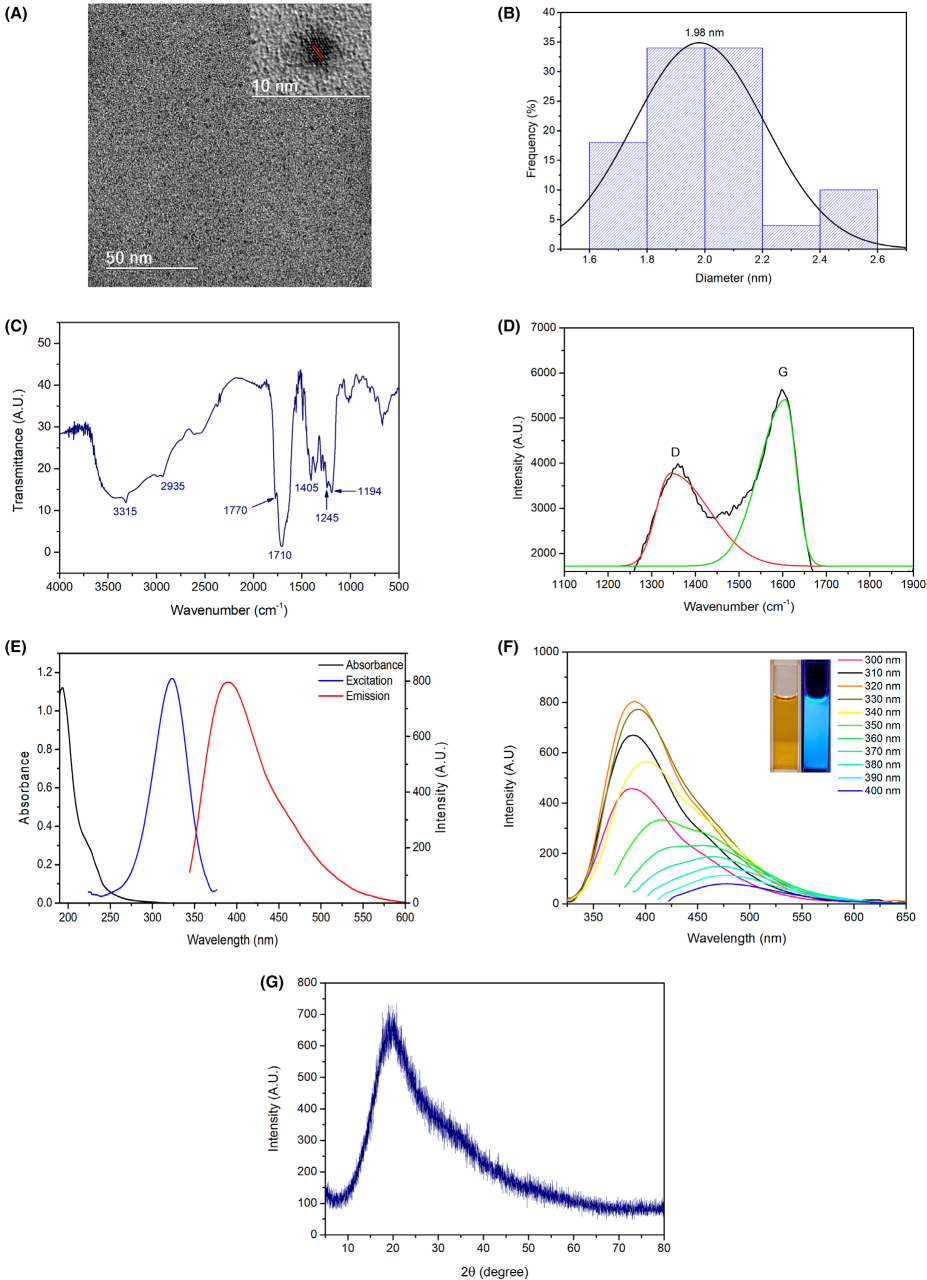
(A) Transmission electron microscopy and high‐resolution transmission electron microscopy for the CD sample; (B): Histogram obtained with a Gaussian fitting curve; (C) Infrared spectrum of CD; (D) Raman spectrum of CD with deconvoluted peaks; (E) Absorption spectra (black line), excitation spectra (blue line) with emission in 391 nm and emission spectra (red line) with excitation in 320 nm for CD sample; (F) Emission spectra under various excitation wavelengths. The inset shows the CD dispersion under both visible and UV light sources; (G) XRD pattern for CD.

Figure 1A shows the transmission electron microscopy (TEM) and high‐resolution transmission electron microscopy (HRTEM) images obtained from a diluted aqueous suspension of the CD deposited on an ultrathin carbon film. By measuring the size of CD from different images (using ImageJ software), it was possible to determine the average size and size distribution of the nanoparticles.

The histogram obtained with a Gaussian fitting curve is shown in Figure 1B and reveals a size distribution ranging from 1.6 to 2.6 nm, with an average size of 2.0 nm. The inset in Figure 1A exhibits an HRTEM image of the CDs and reveals a lattice spacing distance of 0.22 nm, corresponding to the (100) graphitic planes.^38^

Figure 1C displays the FTIR spectrum for the CD sample. The broad band around 3200–3450 cm^−1^ corresponds to O‐H and N‐H stretching. The signal at 2935 cm^−1^ is assigned to C‐H stretching, whereas the bands at 1770 and 1710 cm^−1^ are attributed to C=O stretching vibrations from carboxylic acid functional groups. The peak at 1405 cm^−1^ is associated with the N‐H stretch of amide, and the signal at 1245 cm^−1^ corresponds to C‐O stretching vibrations. Additionally, the band at 1194 cm^−1^ suggests the presence of C‐N stretching vibrations of amines.^39^ These results evidence the presence of carboxylic acid, amine, and amide groups on the surface of the nanoparticle.

From the Raman spectrum of the CD (Figure 1D), two significant peaks around 1355 and 1579 cm^−1^ can be observed. The first signal corresponds to the D band, which indicates the presence of disordered domains, defects, and functionalization. Conversely, the G band is associated with more structured regions of sp^2^ graphitized carbon.^40^ Additionally, in carbon dots (CD), the ID/IG ratio serves as a metric to estimate the degree of graphitization within their internal structure. Since the intensity of the G band correlates with the graphitic structure, a lower ID/IG value suggests a higher degree of graphitization in the nanoparticles. After the appropriate deconvolution of the Raman peaks, the ID/IG value obtained was 0.71, indicating that the CD sample exhibited a higher degree of graphitization.^30^

To investigate the optical properties of the obtained CD, fluorescence emission and UV–VIS absorption measurements were conducted (Figure 1E). The UV–VIS spectrum shows an absorption band peaking around 320 nm, attributed to *n* → π* transitions of C=O bonds.^37^ In contrast, the fluorescence spectrum exhibits a peak emission at 391 nm upon excitation at 320 nm. Photoluminescence spectra (Figure 1F) were acquired across various excitation wavelengths from 300 to 400 nm. The CD sample exhibits fluorescence emission peaks in the blue region of the spectrum, with the emission maximum varying according to the excitation wavelength. The excitation‐dependent emission behavior arises from the presence of distinct functional groups on the surface of the nanoparticles, generating varied energy levels.^41^

The inset in Figure 1F displays an aqueous dispersion of the CD sample under visible light irradiation (left) and UV radiation (right). The dispersion exhibits a transparent yellow hue under visible light and emits a vivid blue fluorescence when exposed to a UV lamp. In addition, the XRD pattern (Figure 1G) of the CD sample revealed a single diffraction peak, centered at 2θ = 20.0°, corresponding to the (002) lattice spacing of graphitic structures. Thus, the HRTEM, XRD, and Raman results together indicate a significant degree of crystallinity in the CDs.

### Characterization of carbon dot‐porphyrin derivative assemblies

**FIGURE 2:**
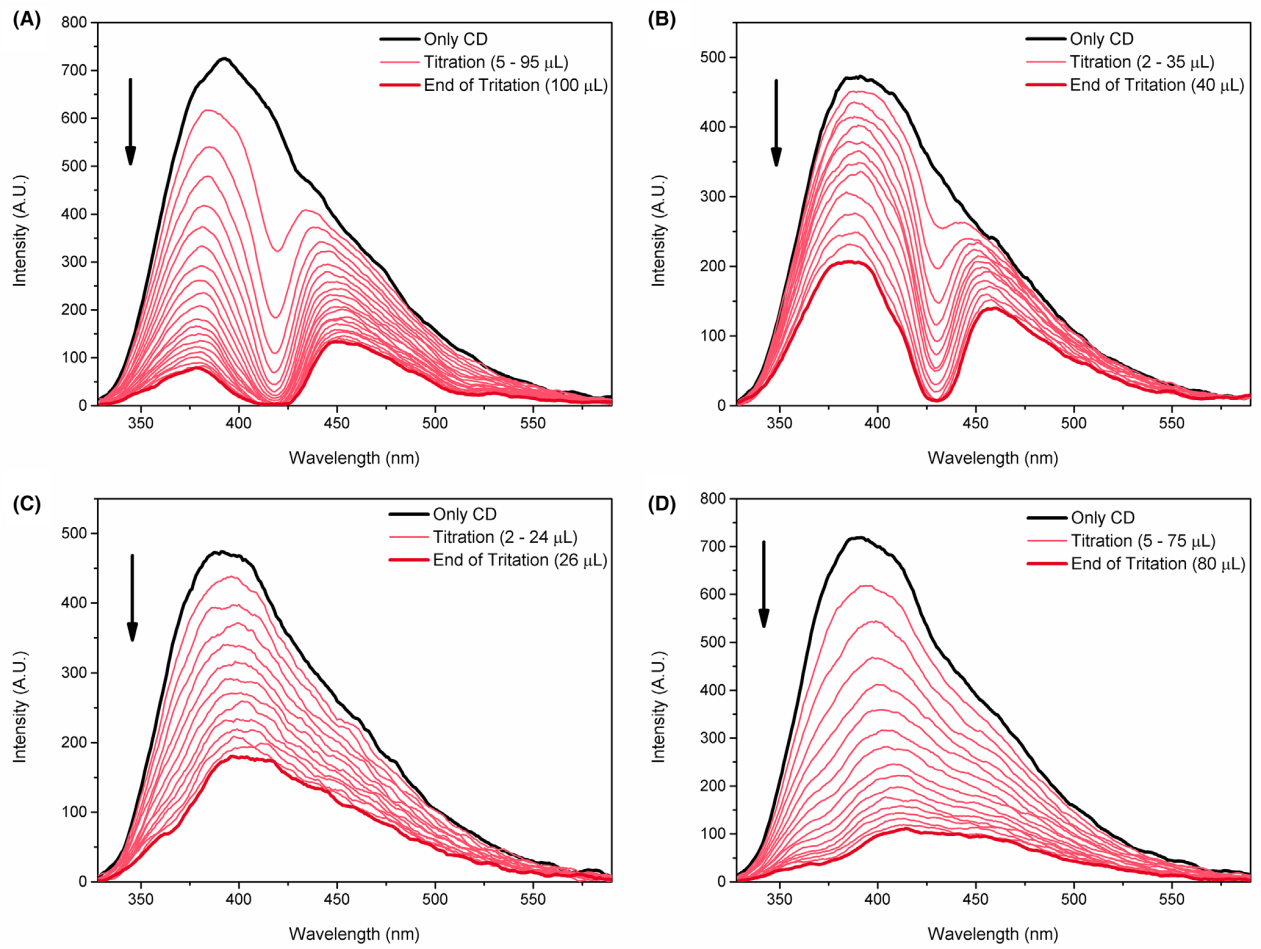
Fluorescence spectra of CD upon titration with H_2_P (A), ZnP (B), H_2_Pz (C), and ZnPz (D) solutions at a concentration of 1 × 10^−3^ mol L^−1^.

To verify the interaction between the porphyrin derivatives (Scheme 1) and the carbon dot, a titration was performed using a fluorescence spectrophotometer, as shown in Figure 2. After each addition of the titrant (porphyrin derivatives), suppression of the CD fluorescence was observed, indicating the existence of an interaction between these substances. This observation was further supported by similar findings reported for another porphyrin in the literature.^22^ The discontinuity observed in the CD band in Figure 2A,B is attributed to the partial absorption of the CD emission by the titrant substances (H_2_P and ZnP), considering that their Soret band falls in this region. Using the titration data (Figure 2), it was possible to determine the Stern–Volmer constants (*K*_SV_) and the intrinsic binding constants (*K*_b_) using Equations ((1), (2), (3)), as shown in Table 1.

**TABLE 1:** Stoichiometric ratio, Stern–Volmer constant (*K*_SV_) and intrinsic binding constant (*K*_b_) of porphyrin derivatives in titration with CD.

Substance	SR^a^ (mol)	*K*_SV_ (×10^4^)	*K*_b_ (×10^5^)
H_2_P	5.17 × 10^−2^	8.5	2.4
ZnP	3.41 × 10^−2^	4.6	0.7
H_2_Pz	2.16 × 10^−2^	9.2	0.6
ZnPz	4.30 × 10^−2^	8.8	6.3

^a^ SR = ratio for 1 miligram of CD.

It is important to mention that Equations (2) and (3) assume that the fluorophore is non‐fluorescent when saturated with the quencher.^42^, ^43^ We could not check the validity of this assumption for our system because the high concentration of porphyrin derivative required to saturate the CD provokes an intense inner filter effect leading to inconclusive results regarding the CD emission. In this sense, we do not intend to compare the values of *K*_sv_ and *K*_b_ from Table 1 with others reported in the literature, but they are still useful for the discussion of some trends found between the compounds used in this study.

The *K*_SV_ demonstrates the ability of a substance to suppress the fluorescence intensity of a fluorophore. Thus, it can be verified that H_2_Pz is the porphyrin derivative that most effectively suppresses the fluorescence of CD. Furthermore, the higher *K*_sv_ values for the free‐base compounds compared to the metallated ones possibly indicate an important role of the macrocycle internal hydrogens in suppressing the emission of CD.

To verify the binding capacity of the porphyrin derivative to CD, *K*_b_ was determined, and high magnitude values (×10^5^) were observed, indicating a strong interaction between the substances. An opposite trend was noted for the compound classes: while porphyrins showed a decrease in *K*_b_ after metalation, porphyrazines exhibited an approximately tenfold increase.

Porphyrin derivatives substituted with phenyl groups have an approximate size of 1.5 nm,^44^ which is relatively large compared to the carbon nanoparticle average size of 2 nm inferred from the TEM analysis. In this sense, a 1:1 ratio is expected for these assemblies to minimize steric and electrostatic repulsion in the structure.

Characterization of the assemblies was further studied by zeta potential (ZP) measurements. Figure S8 shows the ZP values of the free and combined CD suspensions. It can be observed that CD has a net charge close to zero. Despite the carboxylic groups on the surface of CD, the presence of amino groups in acidic medium may contribute to a charge balance. After combination with the porphyrin derivatives, there was an increase of ca. 5–15 mV in the charge of the nanoparticles, indicating that the positive charges of the porphyrin derivatives impact the charge of the nanoparticles. This phenomenon has already been observed for two other porphyrins in the literature.^21^, ^22^ Wu et al.^21^ and Wang et al.^22^ observed that the carbon dots initially exhibited a negative ZP (−15.6^21^ and −17 mV^22^) and became positive after combination (+4.5^21^ and +5 mV^22^).

^1^H NMR analysis of pure H_2_P and H_2_P after the addition of CD can be seen in Figure S9. In the 8–10 ppm region, two of the four aromatic signals of H_2_P undergo significant chemical shifts ranging from 9.86 to 9.79 ppm and from 8.54 to 8.56 ppm upon combination with CD, which is evidence of interaction between these species. When DOSY analysis was performed for these samples (Figure S10), it was found that the translational diffusion coefficient (D) of both H_2_P and CD decreased when combined, thus proving further evidence that a larger structure (assembly) is formed by mixing the porphyrin derivative and the carbon dots.

Another factor that contributes to understanding the interaction mechanism between CD and a quencher is determining whether the interaction is dynamic or static. From a titration with temperature variation (10, 20, and 30°C) (Figure S6), it was observed that there was no change in the concentration versus F/F_0_ relationship for all porphyrin derivatives. Considering that a dynamic quenching mechanism would be favored by the increase in temperature, these results suggest an implicit static interaction mechanism, which is additional evidence for the strong binding between the macrocycles and nanoparticles in the assemblies. This mechanism was also confirmed by analyzing the fluorescence lifetime (Table 2), since after combining the substances there was no change in the fluorescence lifetime of the CD.

**TABLE 2:** Fluorescence decay parameters (lifetimes (*τ*), pre‐exponential factor (A), average lifetime (*τ*_ave_), and chi‐squared (*χ* ^2^)) obtained from the isolated species CD and their assemblies with excitation at 298 nm in water.

Substance	*τ*_1_ (ns)	*Α*_1_ (%)	*τ*_2_ (ns)	*Α*_2_(%)	*τ*_3_ (ns)	*Α*_3_ (%)	*τ*_ave_ (ns)	*χ* ^2^
CD	3.14	33.5	8.41	22.7	0.61	43.8	3.23	1.14
H_2_P/CD	8.45	23.0	3.09	34.9	0.59	42.1	3.27	1.09
ZnP/CD	8.34	23.4	3.06	34.3	0.60	42.3	3.26	1.10
H_2_Pz/CD	8.48	22.3	3.15	34.6	0.61	43.1	3.24	1.15
ZnPz/CD	8.32	23.2	3.02	34.4	0.58	42.4	3.22	1.08

### Photophysical properties of carbon dot‐porphyrin derivative assemblies

**FIGURE 3:**
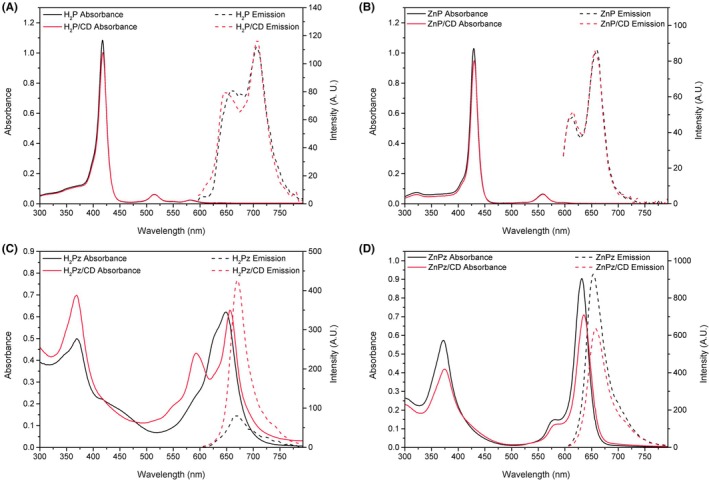
Absorption spectra (solid line) and emission spectra (dotted line) in the UV–VIS region for the isolated porphyrin derivatives (black) and the CD/porphyrin derivatives assemblies (red). (A) H_2_P; (B) ZnP; (C) H_2_Pz; (D) ZnPz.

The formation of the assemblies also impacted the absorbance spectra of the porphyrin derivatives. As shown in Figure 3, there was a decrease in the absorbance of the highest intensity bands of H_2_P (Figure 3A), ZnP (Figure 3B), and ZnPz (Figure 3D) upon combination with CD. A similar decrease in absorbance has also been reported for other porphyrin derivatives in the literature,^23^, ^45^, ^46^, ^47^ indicating a perturbation of the electronic structure of the macrocycles by changes in the microenvironment imposed by the CD. H_2_Pz (Figure 3C) demonstrated a splitting of its Q band when combined with the CD, a phenomenon we have previously observed for this compound in acidic solutions.^25^ Therefore, the spectral changes may be attributed to the acidity of the nanoparticle surface conferred by the carboxylic acid groups.

No significant shifts were observed in the emission bands of the porphyrin derivatives upon combination. However, changes in the emission efficiencies were observed, especially for the porphyrazines, as indicated by the fluorescence quantum yield (*Φ*_F_) values presented in Table 3. H_2_Pz, which shows a *Φ*_F_ of 0.81% in its pure form, showed a significant increase in *Φ*_F_ after combination, reaching a value of 1.97%. In contrast, ZnPz exhibited the opposite effect, with *Φ*_F_ decreasing from 9.3% to 7.6% after combination. Although a fluorescence suppression was described by other groups for zinc phthalocyanines after combination with carbon dots,^48^, ^49^, ^50^ the explanation for such an effect is still unclear, highlighting the need for further investigation with experimental techniques and theoretical calculations.

**TABLE 3:** Fluorescence (*Φ*_F_) and singlet oxygen (*Φ*_Δ_) quantum yields in water of the isolated species and their assemblies.

Substance	*Φ*_Δ_ (%)	*Φ*_F_ (%)
H_2_P	17 ± 2^a^	2.6 ± 0.2^c^
H_2_P/CD	29 ± 1^a^	2.6 ± 0.2^c^
ZnP	44 ± 5^a^	1.20 ± 0.05^c^
ZnP/CD	44 ± 6^a^	1.13 ± 0.03^c^
H_2_Pz	<0.5^b^	0.81 ± 0.03^d^
H_2_Pz/CD	<0.5^b^	1.97 ± 0.05^d^
ZnPz	0.6 ± 0.4^b^	9.3 ± 0.1^d^
ZnPz/CD	1.1 ± 0.3^b^	7.6 ± 0.1^d^
CD	0^a^, ^b^	18^e^

^a^ Irradiation at 532 nm.

^b^ Irradiation at 648 nm.

^c^ Excitation at 577 nm.

^d^ Excitation at 590 nm.

^e^ Excitation at 340 nm.

**FIGURE 4:**
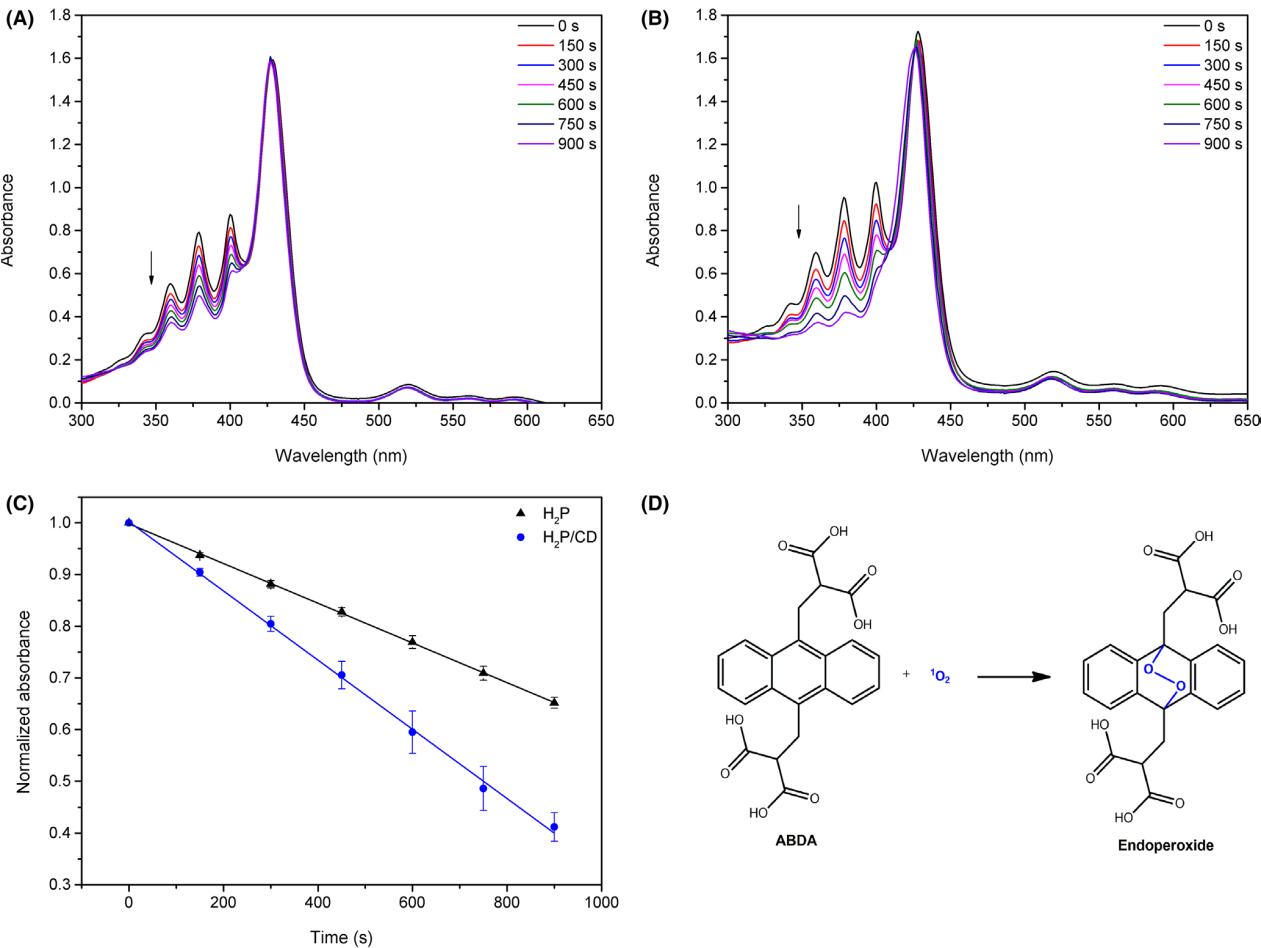
Experiment of *Φ*_Δ_ in water for (A) H_2_P; (B) H_2_P/CD assembly; (C) Kinetics of systems (A) and (B); (D) Chemical equation for the reaction between ABDA and singlet oxygen.

One of the criteria that a photosensitizer (PS) must meet is the production of reactive oxygen species (ROS), primarily singlet oxygen.^6^, ^7^ Ideally, this production should be quantified in an aqueous medium to better simulate the biological medium. Thus, the most widely used probe in an aqueous medium for singlet oxygen experiments is 9,10‐anthracenediyl‐bismethylenedimalonic acid (ABDA), due to its high solubility in water and specificity to singlet oxygen (^1^O_2_), eliminating interference from other ROS.^51^ Basically, when ABDA interacts with ^1^O_2_, an endoperoxide is formed (Figure 4D) that does not absorb in the same spectral region as ABDA. This allows for the indirect measurement of ^1^O_2_ production by monitoring the decay of the absorbance bands at 378 and 399 nm of ABDA.^51^

The obtained values of *Φ*_Δ_ are shown in Table 3, and Figure 4 presents the *Φ*_Δ_ experiment in water for H_2_P (A), H_2_P/CD assembly (B) and the kinetics (C) of the decay of the absorbance of ABDA at 378 nm for these two systems. It was observed that the H_2_P/CD assembly resulted in a greater decay of the absorbance of the ^1^O_2_ probe over the same period as the system containing only H_2_P, consequently leading to a higher *Φ*_Δ_.

A *Φ*_Δ_ of 17 ± 2% was observed for H_2_P and 29 ± 1% for the H_2_P/CD system, indicating that the combination enhances the photosensitizer's performance by approximately 70% after the combination of H_2_P with CD nanoparticles. Some studies in the literature have also shown that porphyrin derivatives demonstrate an increase in ^1^O_2_ production kinetics after the combination with CD,^21^, ^22^, ^46^, ^49^, ^52^ but most of them do not quantify singlet oxygen production (*Φ*_Δ_).^21^, ^22^, ^52^ The studies that show *Φ*_Δ_ values reported increases in singlet oxygen production falling in the range of 12%–20%.^46^, ^49^

The observed increase in *Φ*_Δ_ following combination is likely attributed to an enhanced intersystem crossing to the triplet state, possibly facilitated by the electron‐donating groups present in CD.^53^

Unlike its free base, ZnP did not show the same effect, obtaining a similar *Φ*_Δ_ for the ZnP/CD assembly system, a result also observed for zinc phthalocyanine after combination with CD.^50^ The porphyrazines H_2_Pz and ZnPz demonstrated very low ^1^O_2_ production in water, something also reported for other cationic porphyrazines in the literature,^54^, ^55^, ^56^ which did not significantly change after combination.

## CONCLUSIONS

In this study, we successfully synthesized a carbon dot (CD) from citric acid and ammonium citrate, and assembled them with cationic porphyrins and porphyrazines, as well as their respective zinc complexes. The assemblies were characterized using UV–VIS absorption, fluorescence emission, zeta potential, and nuclear magnetic resonance measurements. Our findings revealed significant changes in the photophysical properties of the combined systems compared to the isolated molecules. After the addition of carbon dots, all compounds demonstrated a decrease in their light absorption, the porphyrazines showed a variation in their fluorescence emission, and the free base porphyrin had a 70% increase in its singlet oxygen production. Furthermore, there was a subtle increase in zeta potential, demonstrating a change in surface charge upon combination.

Overall, the results demonstrate that the combination of CD with porphyrin derivatives can result in significant changes in the photophysical properties of these substances, enhancing their potential as photosensitizers. Thus, these CD/porphyrin assemblies have promising potential for applications in photodynamic therapy, providing a basis for further exploration and optimization of nanoparticle‐based photosensitizers in medical treatments.

## CONFLICT OF INTEREST STATEMENT

The authors declare no conflicts of interest.

## Supporting information


Data S1.

## Data Availability

The data that support the findings of this study are available from the corresponding author upon reasonable request.
