# Supplementary material for: Enhanced fluorescence emission or singlet oxygen production of cationic porphyrazines and porphyrins through combination with carbon dots
Source: Photochem Photobiol. 2025 May 21;101(4):987–99. doi: 10.1111/php.14119 (PMC12345398; doi:10.1111/php.14119)
Supplement: Supplementary file 1 — Data S1. [file PHP-101-987-s001.docx]

**Supplementary Material**

**Enhanced Fluorescence Emission or Singlet Oxygen Production of Cationic Porphyrazines and Porphyrins through Combination with Carbon Dots**

Gustavo Wander Streit, Rafael Bernardino Rodrigues da Silva Taques, Gabriela Fernandes Barreto, Fabiano Vargas Pereira, Gilson DeFreitas-Silva, Thiago Teixeira Tasso

*Chemistry Department, Institute of Exact Sciences, Universidade Federal de Minas Gerais, Belo Horizonte, Brazil.*

***Corresponding author:**

Prof. Thiago Teixeira Tasso

Tel.: +55 31 3409 4987

E-mail address: thiagotasso@ufmg.br


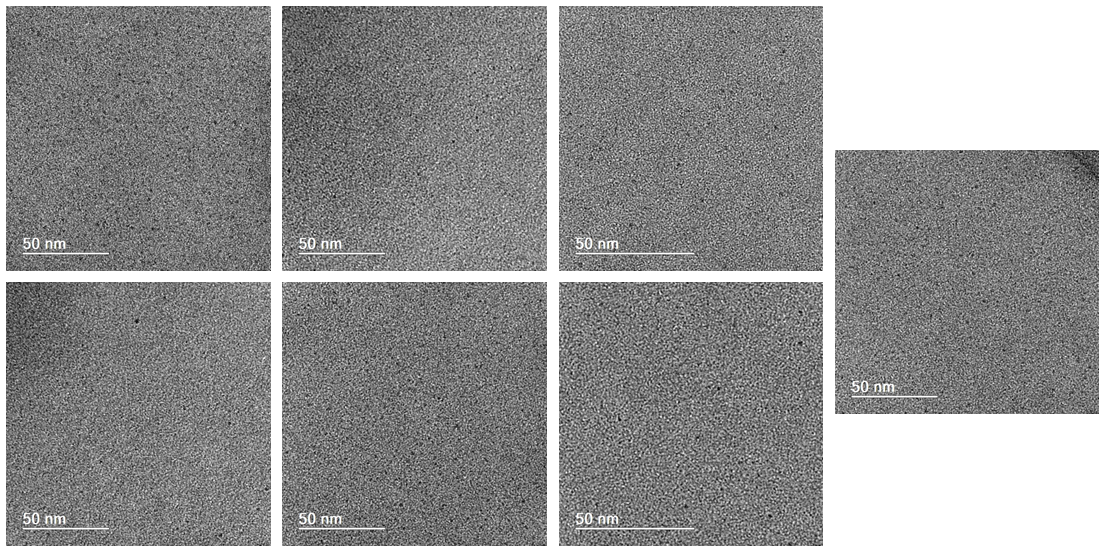


Figure S1 – Images obtained by transmission electron microscopy technique from a diluted aqueous suspension of the CDs deposited on an ultrathin carbon film.


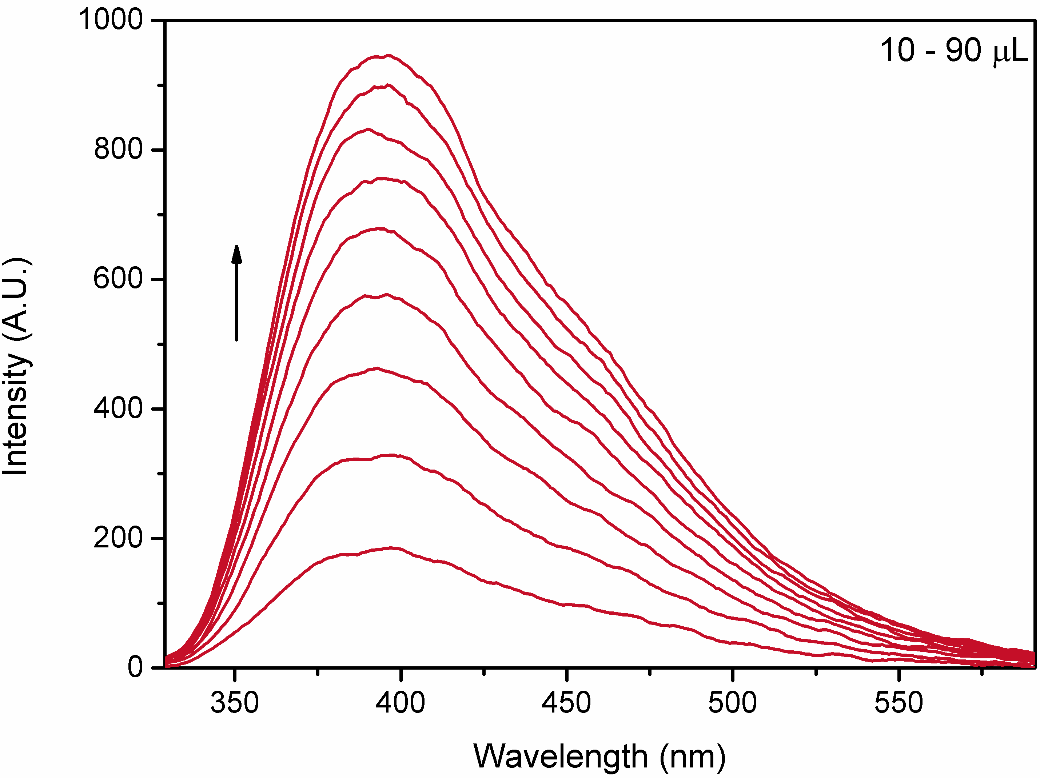


Figure S2 - Variation of emission intensity as a function of CD concentration to determine the value of κ = 1354 ± 12 L.g^-1^.cm^-1^.


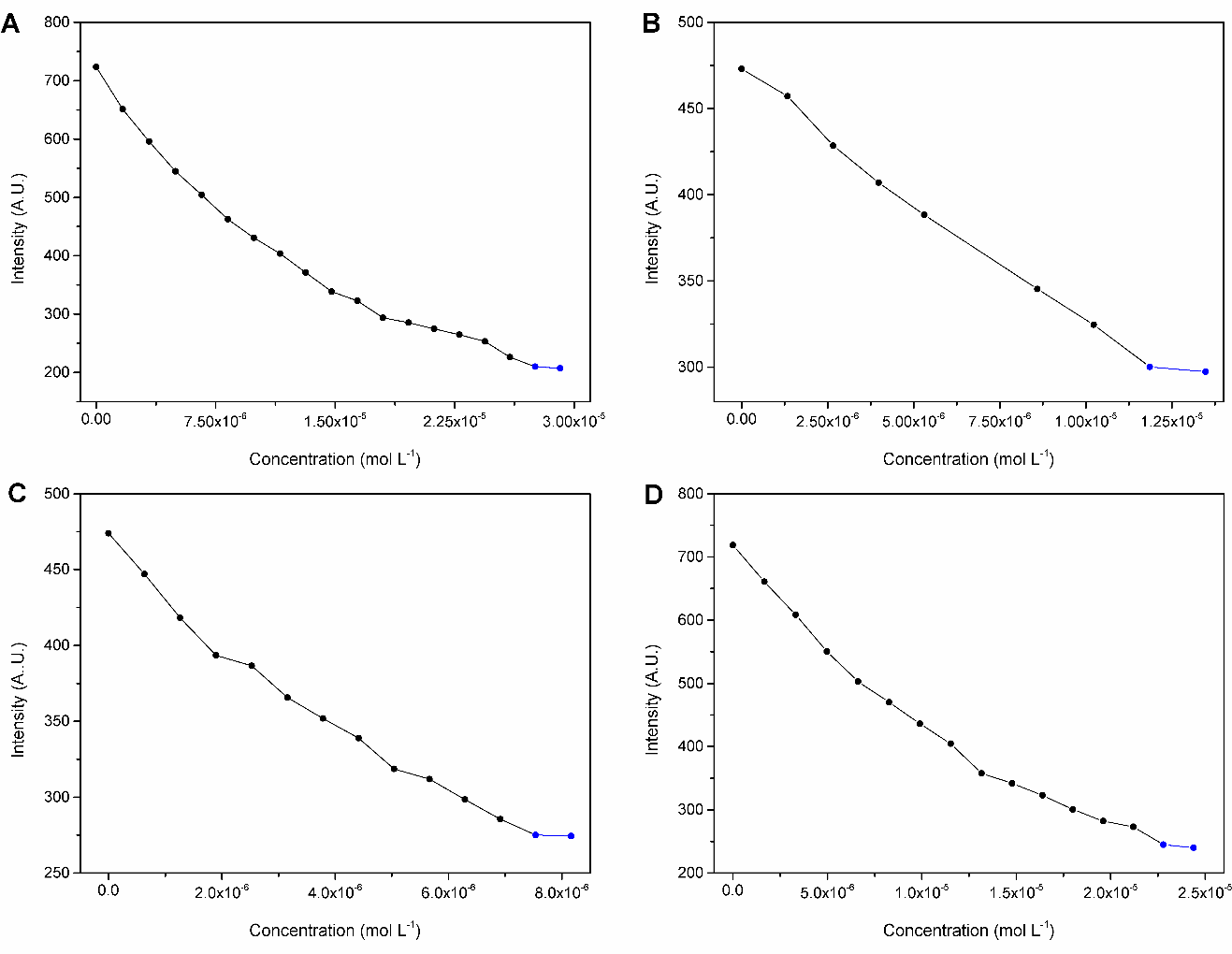


Figure S3 - Concentration of porphyrin derivative added in function of CD intensity. A: H_2_P; B: ZnP; C: H_2_Pz; D: ZnPz.


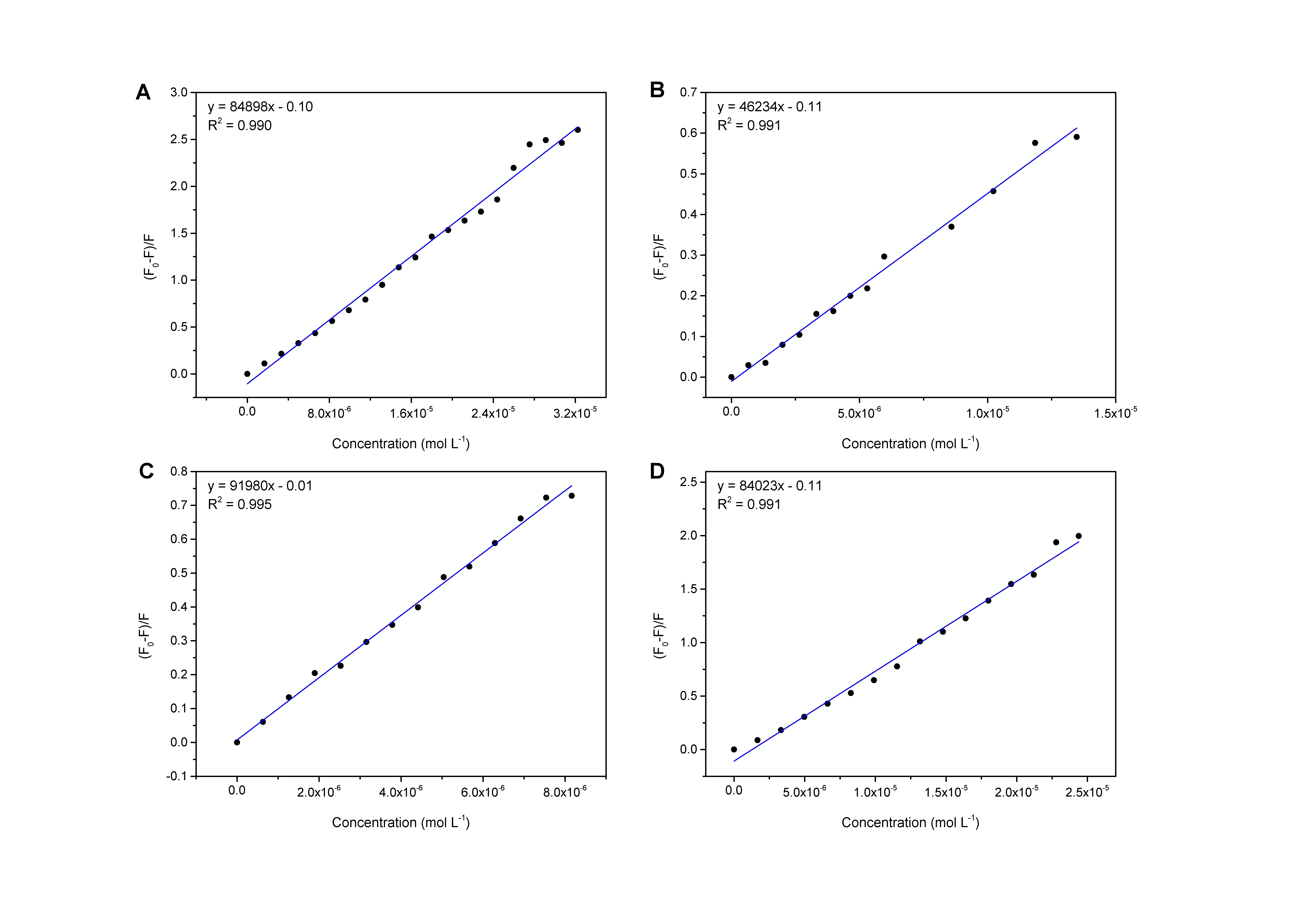


Figure S4 – Concentration of porphyrin derivative in function (F_0_-F)/F for determination of the Stern-Volmer constant (K_sv_). A: H_2_P; B: ZnP; C: H_2_Pz; D: ZnPz


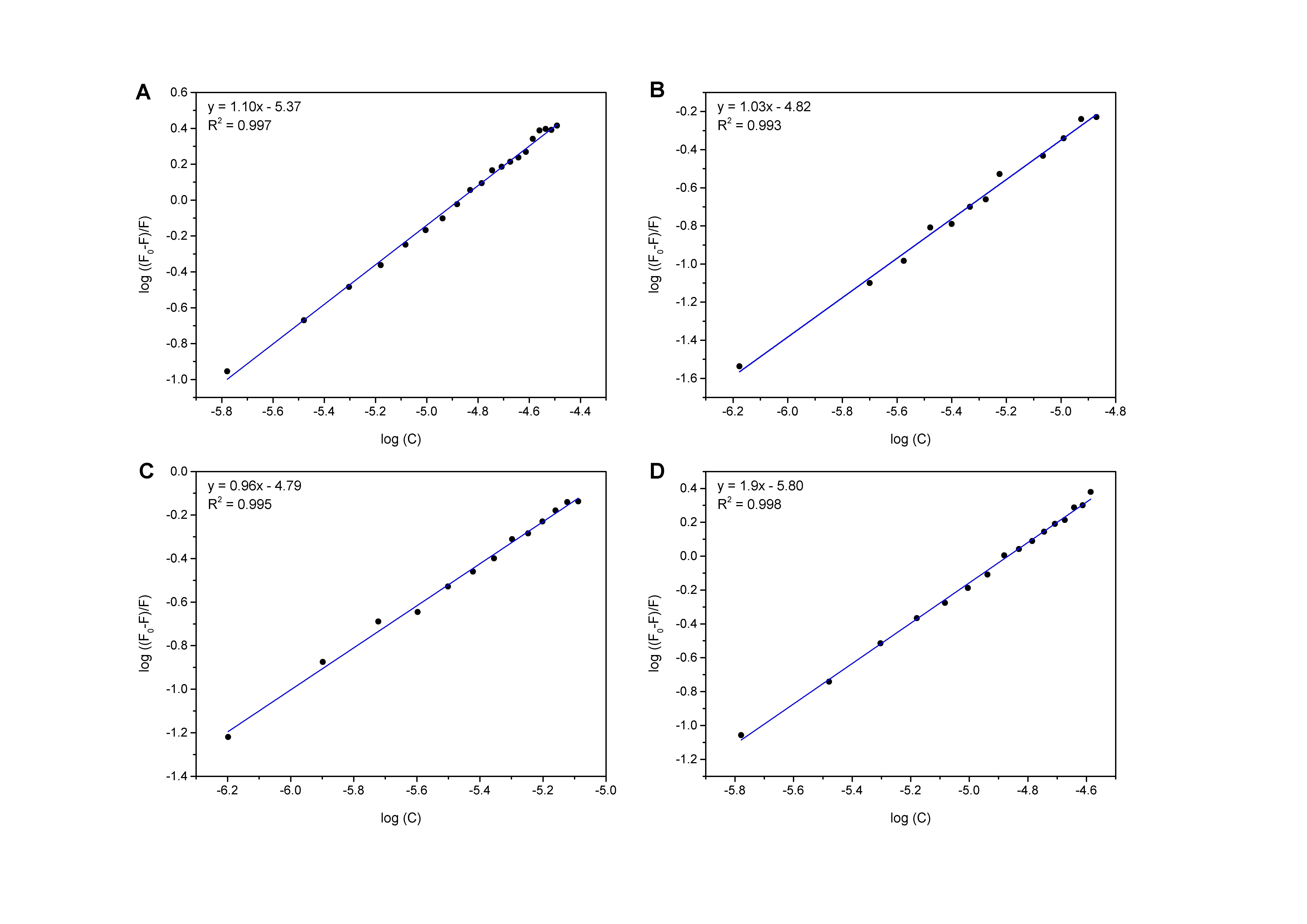


Figure S5 - Graph to determine the intrinsic binding constant, plotted by log of the concentration of porphyrin derivative vs log of (F_0_-F)/F. A: H_2_P; B: ZnP; C: H_2_Pz; D: ZnPz.





Figure S6 – Evaluation of the interaction between CD and the porphyrin derivatives varying the temperature (10, 20 and 30 ^o^C). A: H_2_P; B: ZnP; C: H_2_Pz; D: ZnPz.


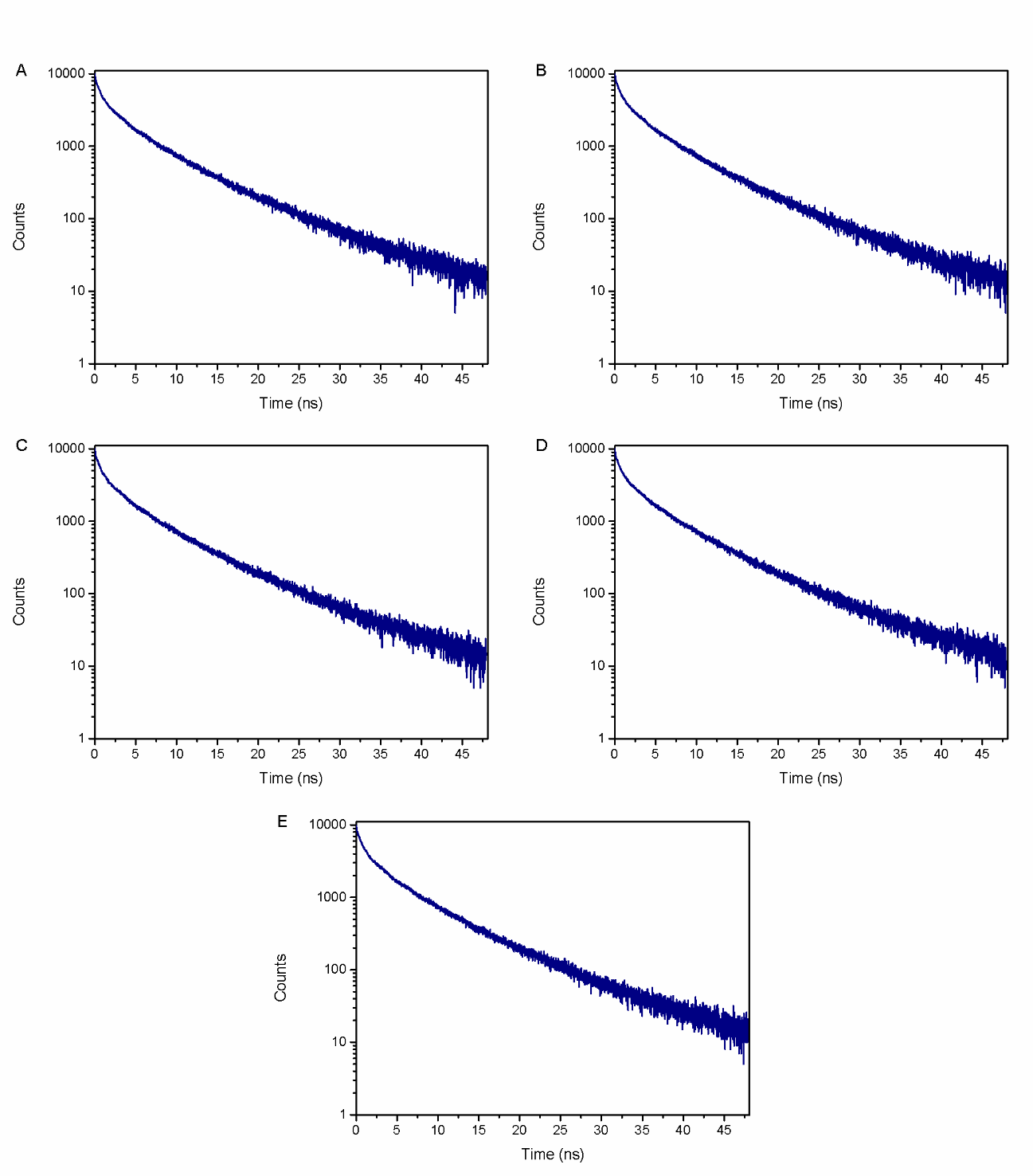


Figure S7 – Fluorescence lifetime decays curves for A: H_2_P/CD; B: ZnP/CD; C: H_2_Pz/CD; D: ZnPz/CD; E: CD.


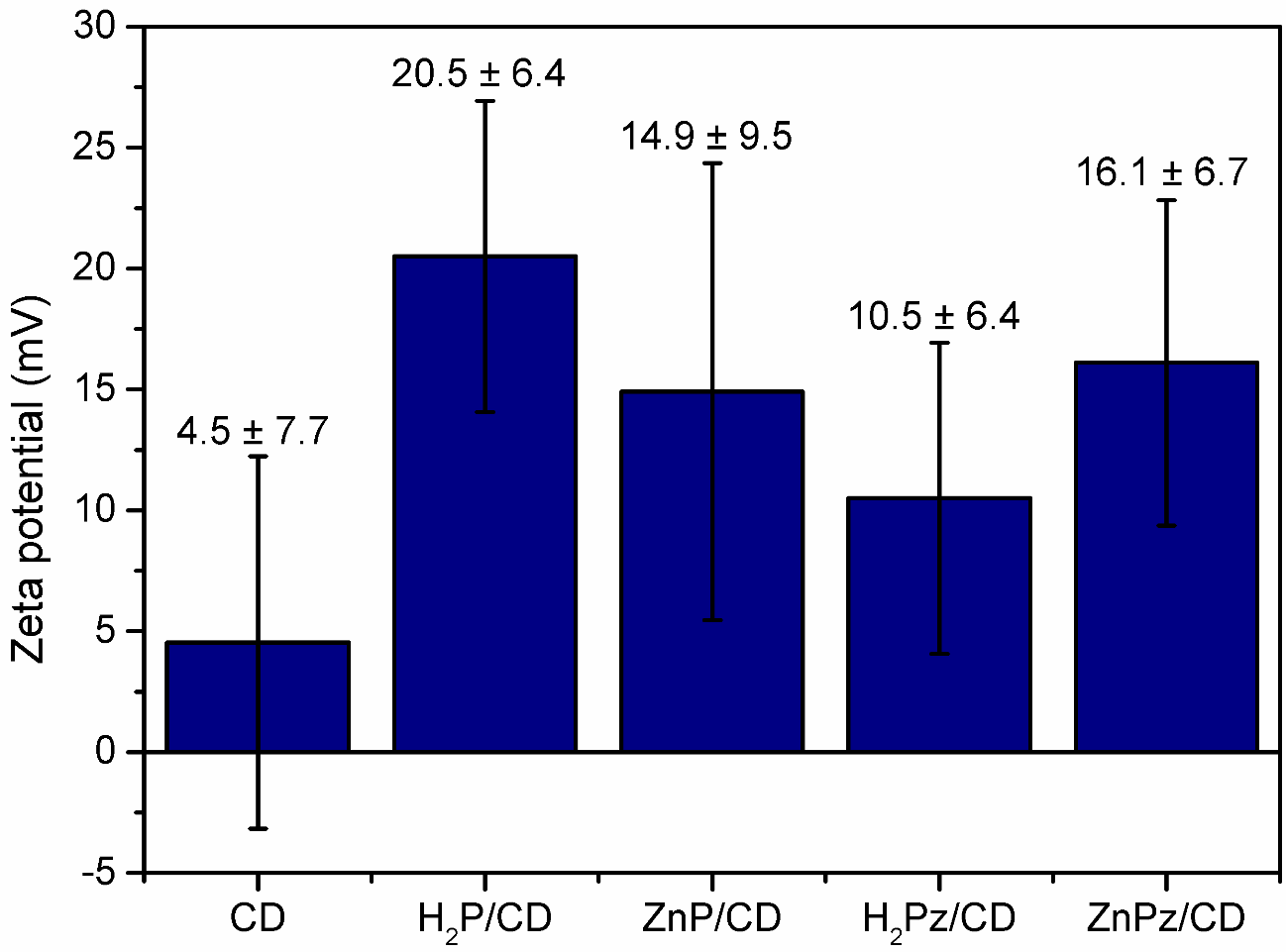


Figure S8 - Zeta potential of CD and porphyrin derivatives assemblies to CD.


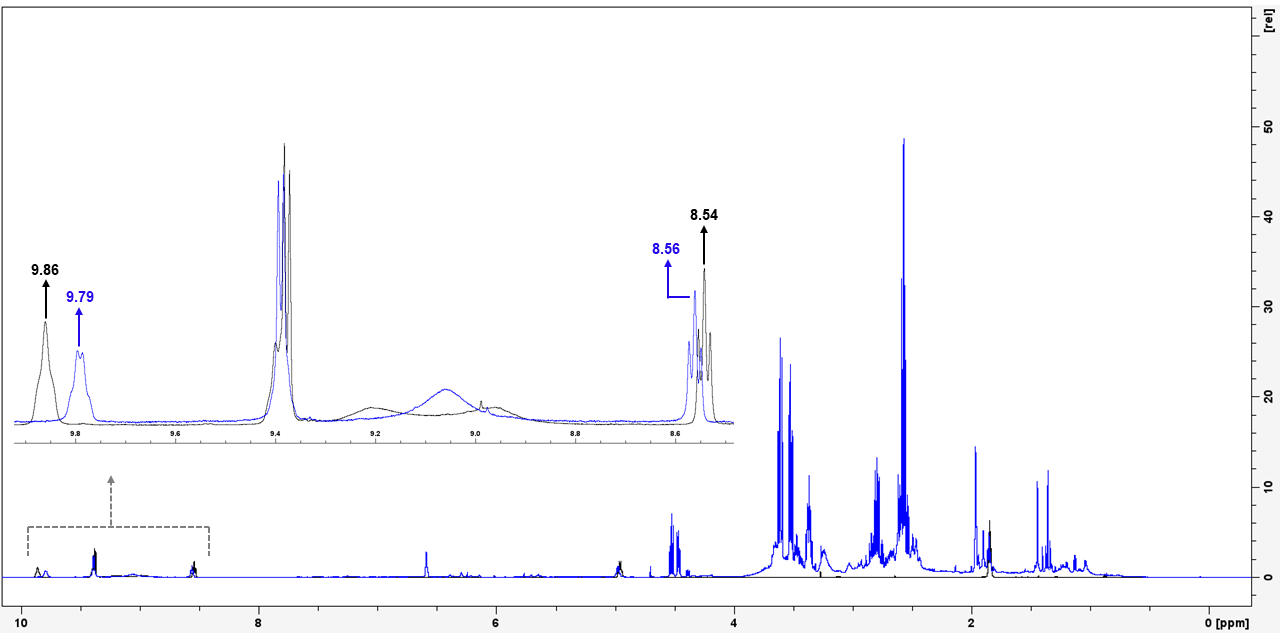


Figure S9 - ^1^H NMR in D_2_O with pre-saturation of the 4.70 ppm signal (water in D_2_O) of pure H_2_P (black) and the H_2_P/CD assembly (blue).


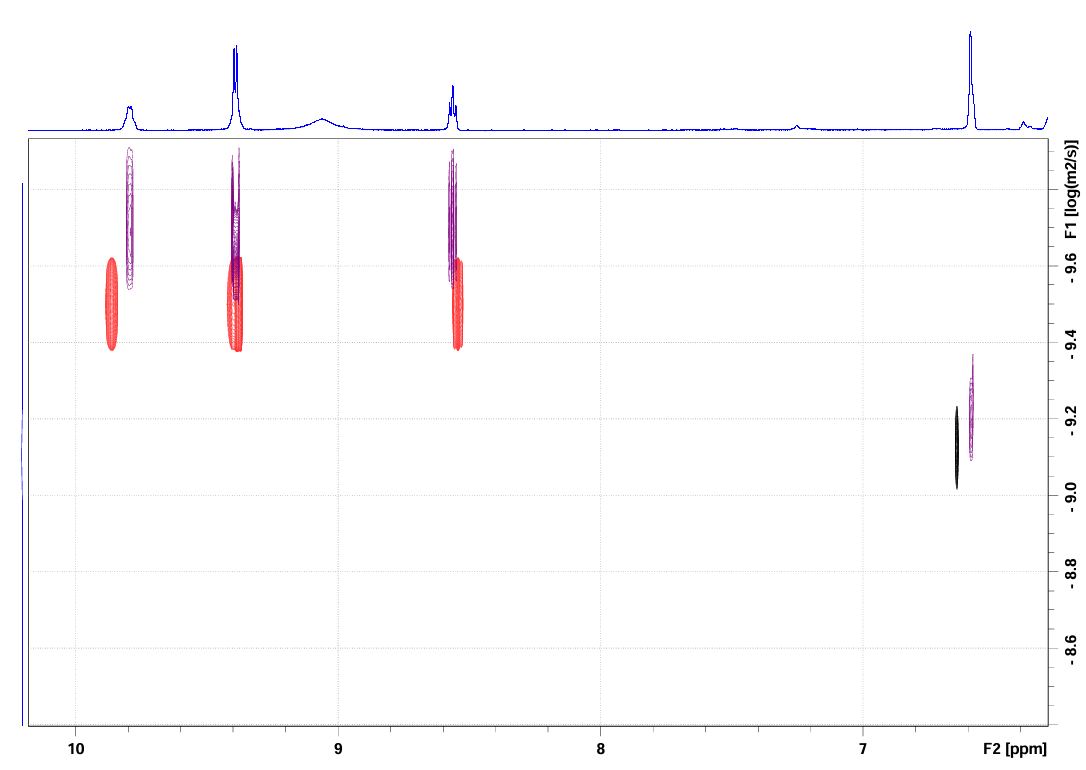

Figure S10 - DOSY NMR spectrum obtained for H_2_P (red), CD (black), and H_2_P/CD (purple) samples. The translational diffusion coefficients (D) were determined for representative signals of the samples, shown on a logarithmic scale (F1: Log D [m²/s]) *versus* chemical shift (F2, ppm). The experiment was performed using the stebpgp1s19 pulse sequence, with the following parameters: number of scans (ns) = 32, number of acquired points (TD) = 4k, number of diffusion points = 16, and diffusion curve type = linear.
